# COMPUTER SECURITY—FOR TODAY… AND FOR TOMORROW

**DOI:** 10.6028/jres.092.006

**Published:** 1987-02-01

**Authors:** Irene E. Isaac

**Affiliations:** Computer Security Management and Evaluation Group, Institute for Computer Sciences and Technology, National Bureau of Standards, Gaithersburg, MD 20899

The Institute for Computer Sciences and Technology (ICST) and Department of Defense (DoD) National Computer Security Center (NCSC) jointly sponsored the Ninth National Computer Security Conference held at the National Bureau of Standards. The September 1986 conference attracted more than 800 people from government, industry, and academe eager to share information and to learn of new approaches and future trends in “trusted” computer systems. A “trusted” computer system is one that employs sufficient hardware and software integrity measures to allow simultaneous processing of multiple levels of classified or sensitive information. Stated another way, the term “trusted” computer system simply means we can rely on the computer itself to protect information from unauthorized use or modification.

Under the theme “Computer Security—for Today … and for Tomorrow,” the conference provided a forum for technology interchange among developers of trusted systems and a place where computer users could exchange ideas and learn of new ways to apply some of the current computer and information security technology.

## Background

Computers were first used to solve our information processing and dissemination problems. Security design principles, in general, were not used in building most commercial computer systems. This vulnerability, coupled with large amounts of highly sensitive and classified information processed by computers, resulted in an awareness that the security objectives of maintaining confidentiality, integrity, and availability of computer systems could not be assured.

Through its Computer Security and Risk Management Program, the Institute for Computer Sciences and Technology has played a vital and unique role in helping to protect government computer systems from intentional and accidental destructive acts. The activities of the program were established in 1965 by the Brooks Act and affirmed in 1980 by the Paperwork Reduction Act. Since 1972, ICST has issued cost-effective standards and guidelines for protecting computerized information from numerous threats including human error, natural hazards, and unauthorized users. The program encompasses research and development of security standards, test methods, transfer of technology to potential implementors and vendors, and technical assistance to advance new uses of computer technology. To carry out its computer security program, ICST researchers work cooperatively with a broad spectrum of organizations from Federal, State, and local governments; industry computer users and manufacturers; research organizations; and voluntary standards groups. While ICST’s computer security program primarily assists Federal agencies in meeting their computer security responsibilities, the private sector is making increasing use of the program’s services and resources.

Still faced with a lack of trusted computer systems upon which to depend in multiple classification processing environments, DoD organizations adopted a policy whereby system access was denied to all except those cleared to the highest level of classification of any information contained in the computer system. Enforcing such policy, however, required considerable duplication of resources and placed a strain on security clearance procedures.

In 1978 the Assistant Secretary of Defense for Communications, Command and Control and Intelligence established the DoD Computer Security Initiative to achieve widespread availability of trusted computer systems for use within DoD. This initiative was intended to foster development of computers by industry that would provide a high degree of integrity for protecting sensitive and classified information.

Since the start of the DoD Computer Security Initiative, industry has met the challenge of the security problem. Several trusted computer systems have been developed for use within the DoD. Evaluation procedures have been established for determining the environments for which a particular trusted system is suitable. The “Department of Defense Trusted Computer System Evaluation Criteria,” published August 15, 1983, establishes a uniform set of basic requirements and evaluation classes for accessing the effectiveness of security controls built into computer systems. This document is commonly referred to as the “Orange Book.” The evaluation criteria, as defined in the Orange Book, are divided into four broad hierarchical divisions representing enhanced security protection: D, C, B, and A. Each division represents a major improvement of security controls found in a computer system. The highest division “A” is reserved for systems providing the most comprehensive security. Furthermore, the problems of computer security have gained greater importance and attention in government at all levels and in business. In recent years, the initiative has been expanded to encourage research that is responsive to a broad class of computer security needs. Computer manufacturers continue research for the development of additional trusted systems that can be effectively used by not only the DoD, but civil agencies and the private sector.

The Technology Transfer Program continues to be an important element of the DoD Computer Security Initiative. In 1979, ICST joined with DoD to assist in stimulating a higher awareness of both the information security problems and solutions. Mr. Stephen Walker, who was then Chairman of the Computer Security Technical Consortium and Dr. Dennis Branstad, ICST, (now an NBS Fellow), organized the first of a series of meetings that would become an invaluable source for information exchange.

Since that first meeting held at the National Bureau of Standards in 1979, attendance and active participation have grown, and meetings have progressed from seminar to symposium to conference. The growth in participation represents an increase in information security awareness as well as a parallel maturation of the technology and its use. The combined efforts of ICST and DoD have brought about much of this progress.

## About the 1986 Conference

Two parallel tracks, one of which addressed managerial computer security issues and the other technical issues, allowed conferees to focus on areas that were of interest or visit sessions in both categories to gain a broad perspective of trends in securing information systems. While many of the papers presented at this conference addressed issues relevant to trusted computer systems designed to support military computer applications, topics which dealt with a broader and more practical utilization of computer security technology were also discussed. Some of the management issues explored included education and awareness training, risk management, contingency planning, and computer security auditing. Some of the technical issues discussed included secure operating systems, security models, verification techniques, database security, and network security.

Speakers for both tracks included computer industry leaders, computer security practitioners and researchers from the United States and abroad. Brief summaries of a few specific contributions follow.

### Opening the Conference

ICST Director, James Burrows and NCSC Director, Patrick Gallagher welcomed the conference participants. Mr. Burrows stressed the need for alliances between users and suppliers to meet future needs for secure applications. Mr. Gallagher discussed NCSC activities and called for cooperative efforts from the government, industry, and academe to solve security problems.

The keynote speaker, Walter S. Harner, Director of Complex Systems Technology for International Business Machines (IBM), said that industry had made progress in developing computer systems with security features but that there were still many issues to be addressed. He called for uniform standards for secure systems with extensions defined for special applications such as national security.

### Database Issues

Several papers dealt with the integrity issue for trusted database systems. Each addressed the inference and aggregation problems as well as other security threats. Inference occurs when the user is able to infer some fact from the information that has been presented. Aggregation occurs when data combined from different sources results in a data item that has a higher classification than its individual components.

Rhonda Henning and Swen Walker, both from NCSC, presented a joint paper with Ms. Henning reporting on various system configurations which support database management systems (DBMS) and the security tradeoffs inherent in each. She pointed out that the incorporation of security features into a commercial DBMS is not an easy task, that a trusted DBMS will depend upon the careful inclusion of appropriate security controls, a sufficient audit trail, and a thorough recovery capability. She concluded that problems vital to database security were not fully understood and that only when these issues were properly addressed could DBMS be considered secure. She suggested that building prototype database management systems could provide insights into the problem.

Similarly, Roger Schell, Gemini Computers, Inc., and Dorothy Denning, SRI International, presented a joint paper which addressed integrity issues essential to the operation of secure database systems. Dr. Schell discussed all aspects of mandatory (classification/clearance) and discretionary (need-to-know) integrity controls both of which can protect data from malicious tampering and destruction as well as from accidental modification and destruction. Dr. Schell pointed out that database integrity rules should be included in an overall integrity policy because they provide users with considerable assurance that the data can be protected against many errors.

Peter Troxell, PAR Government Systems Corporation, discussed the hardware and software components which must be present in a “trusted” DBMS. He presented two approaches, Views and Integrity-Lock, both of which have been proposed as solutions to the trusted database design. He discussed the strengths and weaknesses of each of the proposed architectures. He concluded that further work must be done on how to implement the discretionary security policy onto a database.

### Toward Improving Operational Systems

Many claims have been made about the degree of protection afforded by dial-up communications security devices. Gene Troy, ICST, reviewed a wide variety of hardware devices that are on the market. He discussed the limitations of these dial-up security devices, pointing out their flaws and implementation weaknesses. Mr. Troy recommended a series of approaches to overcoming these problems.

Also on the topic of improving operational systems, Hal Feinstein, MITRE Corporation, discussed security problems faced by civilian agencies operating unclassified, but sensitive systems. He pointed out that much of the security technology has been developed for the protection of classified information and that this technology cannot be effectively applied to unclassified environments of the Federal Government. Mr. Feinstein reviewed the multi-level sensitivity sharing problem suggesting that the risks can be reduced by good software security engineering and basic security enhancements to the operating system. He emphasized the need to develop a consistent, government-wide classification or ranking system for sensitive information similar to the labeling structure used in military environments. He further suggested that such a classification system would allow managers to more adequately determine the level of protection needed for handling and transmitting sensitive applications and data. Mr. Feinstein proposed a classification system ranging from non-sensitive to extremely sensitive. He pointed out that while these sensitive classifications conform to the military labeling structure, they are without national security implications.

### Verification and Analysis

David Balenson, ICST, described the NBS Message Authentication Code Validation System (MVS) which permits remote, automated testing of systems employing message authentication for conformance to various standards. Mr. Balenson addressed topics which led to the development of the MVS, the standards it validates, its design philosophy, the requirements placed on vendors who wish to validate their devices, performance characteristics, and the results of the validations performed to date. Mr. Balenson explained that security standards have been developed within ICST and the Financial Community to authenticate computer data and electronic financial transactions. He pointed out that these standards, Federal Information Processing Standards Publication (FIPS PUB) 113 and the American National Standards Institute (ANSI) X9.9, make use of the Data Encryption Standard (DES) cryptographic algorithm to calculate a cryptographic checksum or Message Authentication Code (MAC) which is used to detect the accidental or intentional modification of computer data. Miles Smid and Elaine Barker, also from ICST, are co-authors of the paper presented by Mr. Balenson.

F. Javier Thayer, MITRE Corporation, proposed a discipline for verifying software. Three interactive processes were proposed: a modeling process, a theorem-proving process, and a review and acceptance process. Suggestions were made for improving the development of these processes. He recommended that the modeling process should develop formal mathematical models of natural language requirements or specifications. He referred to the modeling-process as the most critical part of verification. In the theorem-proving process, the second process of formal verification, mathematical proofs of the conjectures generated during the modeling process should be constructed and analyzed. He pointed out that the review and acceptance process generally means ascertaining that the verification satisfies requirements agreed upon by the customer and verifier. He emphasized that this review and acceptance process should not only satisfy the requirements set forth by the customer, but should point to the soundness of the principles used in the verification. He called attention to the idea that the review process should allow for interaction between the reviewers and the verifiers. The ideas presented here were principally drawn from a review of the design verification of the Restricted Access Process (RAP). Co-authors of the work presented by Mr. Thayer included Dale Johnson, and William Farmer also of MITRE Corporation.

### Foundations

Pamela Cochrane, Trusted Information Systems, Inc., reported the initial findings of a research project undertaken to investigate the feasibility of creating a trusted version of the Mach-1 operating system being developed at Carnegie-Mellon University. Ms. Cochrane, explained how Accent, the progenitor of Mach-1, is being used to conduct initial analysis, since both systems are message-based and focus on ports (kernel-managed message queues). She explained that her organization’s research was targeted toward a class B3 Trusted Computing Base (TCB). The DoD Trusted Computer Security Evaluation Criteria (TCSEC) defines B3 systems as those highly resistant to penetration. Specifically, B3 systems preserve the integrity of sensitivity labels and use them to enforce a set of mandatory access control rules. Systems with B3 ratings are further characterized by a commitment to satisfy the reference monitor requirement that the system mediate all accesses to objects by subjects, be tamperproof, and be small enough to be subjected to analysis and tests.

Ms. Cochrane discussed two different approaches to labeling that are being probed. These approaches are 1) Kernel Mediation Approach—associating labels with ports and processes managed by the kernel; and 2) Server Mediation Approach—modifying the existing access group structure and using the Authentication, Authorization, and Name Servers to provide mandatory access control. She concluded that current investigations indicate that the Accent system could be modified to generate a viable B3 level operating system, and that the Kernel Mediation Approach, with labeling of all subjects and objects and a minimized TCB, is a strong candidate for B3. Further investigation of both approaches will continue. The research activities discussed in this paper were cooperative efforts of Ms. Cochrane, Martha Branstad, D. Elliot Bell, and Stephen Walker who are staff members of Trusted Information Systems, Inc.

### Vendor Activities

Honeywell Information Systems has the only two commercial products available on the NCSC Evaluated Products Lists above Class C2 (this class enforces individual user accountability through login procedures, auditing of security-related events, and resource isolation). The Multics Product is rated as a class B2 system (provides structured protection); the Secure Communication Processor (SCOMP) was rated A1 (verified protection), the highest rating available.

Lester Fraim, Honeywell, Corp., discussed Honeywell’s strategy for developing future products that will meet high-level security requirements. He informed the audience of the research activities of the Honeywell Secure Computing Technology Center. He spoke of Honeywell’s plans to build on the technology of the SCOMP product, as well as integration of new technology as it becomes available, into sound technical solutions.

## Conclusion

As was evidenced throughout the conference, research activities are aggressively being pursued toward development of technologies that will enhance the integrity and security of our nation’s computing systems and networks. It is widely recognized, however, that a great deal of research and development is needed before a significant number of “trusted” computing systems become available. With continued contributions from Federal Government, private industry, and academe this goal is certain to become a reality.

Proceedings from this conference are available upon request. You may write or call Irene Isaac, ICST, (301) 975-3360. Next year’s conference will be held at the Baltimore Convention Center, Baltimore, MD, so that a larger audience may be accommodated. The conference will be announced in early 1987.

